# Genome-Wide Identification and Characterization of *Aldo-Keto Reductase* (*AKR*) Gene Family in Response to Abiotic Stresses in *Solanum lycopersicum*

**DOI:** 10.3390/ijms24021272

**Published:** 2023-01-09

**Authors:** Xiaoyu Guan, Lei Yu, Aoxue Wang

**Affiliations:** College of Horticulture and Landscape Architecture, Northeast Agricultural University, Harbin 150030, China

**Keywords:** tomato, aldo-keto reductase family, abiotic stresses, VIGS

## Abstract

Tomato is one of the most popular and nutritious vegetables worldwide, but their production and quality are threatened by various stresses in the environment in which they are grown. Thus, the resistance and tolerance of tomatoes to various biotic and abiotic stresses should be improved. Aldo-keto reductases (AKR) are a superfamily of NAD(P)(H)-dependent oxidoreductases that play multiple roles in abiotic and biotic stress defenses by detoxification and reactive oxygen species (ROS) clearance pathways. Here, 28 identified *AKR* family genes of tomatoes were identified genome-wide, and their characteristics, including chromosomal location, gene structures, protein motifs, and system evolution, were analyzed. Furthermore, the phylogenetic and syntenic relationships in *Arabidopsis thaliana*, rice, and tomatoes were compared. Expression patterns at different tissues and in response to abiotic stresses, such as drought and salt, were monitored to further explore the function of *SlAKRs*. Finally, three *SlAKRs* candidate genes were silenced by Virus induced gene silencing (VIGS) systems in *Solanum lycopersicum*, showing sensitivity to drought and salt stresses with low contents of proline (Pro) and peroxidase (POD) and high content of malonaldehyde (MDA). This study provides the characteristics and potential functions of *SlAKRs* in response to abiotic stresses that will be helpful for further studies in *S. lycopersicum*.

## 1. Introduction

Aldo-keto reductase (AKR) is a superfamily of NAD(P)(H)-dependent oxidoreductases, which is composed of many monomer types of protein members [1,2]. At present, more than 190 *AKR* family members have been identified because of the high conservation of the *AKR* family during evolution, and *AKR* members are classified into 16 families on the basis of amino acid sequence similarity, with more than 60% high homology in the same subfamily [2,3]. Each AKR member contains a conserved structural domain of AKR with classical (α/β) 8-barrel fold structures. Moreover, three loops (Loops A, B, and C) are observed at the carboxyl terminal of these structures with size and number differences according to their specificity. However, *AKR* family members have similar three-dimensional structures [4]. *AKRs* have been reported to be widely found in animals and plants involved in the modification of bioactive compounds and detoxification and have a wide range of substrates (such as reduction of sugars, aldehydes, and ketones; hormones; chemical carcinogens; and various carbonyl compounds) [5]. In animals, *AKRs* play multiple roles and are responsible for preventing mammalian cells from the toxic and carcinogenic effects of different genotoxic and nongenotoxic chemicals by reduction [6]. In plants, *AKRs* play roles in metabolic pathways, such as activated aldehyde detoxification, osmotic pressure synthesis, secondary metabolism, and membrane transport, and are reported to have functions in biotic and abiotic stress pathways by regulating secondary metabolism and maintaining cellular osmotic pressure [7,8].

Plants inevitably encounter biotic and abiotic stresses, such as salt and alkali stresses in soil, extreme temperatures (heat or cold), drought, diseases, and insect pests [9]. A large number of REDOX enzymes are activated in plants to be against the superfluous acetaldehyde and reactive oxygen species (ROS), which decompose toxic substances, such as the AKR family involved in the degradation of ROS and active acetaldehyde to detoxification, to resist adverse environment [10,11,12,13,14,15]. With the rapid development of genomics in recent years, *AKR* families are increasingly identified and even cloned in different species of plants, such as *Arabidopsis thaliana* [10], rice [11], *Hordeum vulgare* [12], *Medicago truncatula* [13], and strawberry [14], for further research. *AKR* genes play roles in promoting resistance to stresses in the plant by detoxifying reactive aldehydes and in many redox reactions [8,15]. Bartels et al. found that the AKR4C1 protein in barley has an osmoprotective function during barley embryo development [12]. The heterologous overexpression of *AtAKR4C9* in barley can improve its tolerance in response to salt stresses [8]. *AtKR4C9* is expressed more in the sepals of *A. thaliana* than in other tissues and is induced by the infestation of *Botrytis cinerea* and *Pseudomonas syringae* [10]. The mRNA levels of *PpAKR1* are highly increased by abscisic acid, oxidative stress, and cold and salt stress treatments in *Prunus persica*, and the overexpressed *PpAKR1* of transgenic *A. thaliana* lines increases the tolerance to salt stresses by enhanced NADP-dependent dehydrogenase activity [16]. *OsAKR1* is transformed into tobacco and improves tolerance to high temperatures in tobacco by malondialdehyde and methylglyoxal detoxification [11]. The transgenic *A. thaliana* seedlings of *OsAKR4C15* are more tolerant to stresses compared with wild types due to the induced low contents of Malondialdehyde (MDA) and metabolizing methylglyoxal (MG) [17]. The overexpression of *IbAKR* in tobacco is more tolerant to Cd stresses by clearing cytotoxic aldehydes and ROS clearance [3]. Gavidia et al. found that AKR4C5 and AKR4C6 proteins in digitalis can play roles in the biosynthesis of cardiac glycosides besides their functions in response to abiotic stresses in plants [18]. *FaGalUR*, an *AKR* member of strawberries, plays a role in the biosynthesis of ascorbic acid in the fruits of *Fragaria X ananassa* [19]. In conclusion, *AKR* family members may be widely involved in resistance to stress in the plant.

Tomato, which is derived from South America and widely cultivated in China, is one of the most important thermophilic horticultural plants worldwide and is subjected to a variety of stresses, including salinity, drought, and pathogens [20]. Most modern tomato cultivars, such as *Solanum lycopersicum*, are sensitive to moderate levels of salinity stress, drought stresses, and high temperatures, leading to a 70% loss in yield [21]. Therefore, the resistance and tolerance of tomatoes to various biotic and abiotic stresses should be improved. *AKR* family genes have been reported in rice, *A. thaliana*, and soybean but rarely in tomatoes. Therefore, in this study, the *AKR* gene family members of tomatoes are searched and identified over the whole genome associated with bioinformatic analysis to analyze their gene structures, chromosome distribution and locations, motif sequences, conserved domains, evolutionary relationships, and physicochemical properties. The expression profiles of *AKR* family members in different tissues of tomato, such as root, stem, leaves, flower, and fruit, are detected by RT-PCR. Expression levels are determined under drought and salt stresses at different times to further investigate their regulatory network expression in tomatoes and further understand the function of *SlAKR* family genes.

## 2. Results

### 2.1. Identification and Characteristics of SlAKR in S. lycopersicum

A total of 28 predicted *AKR* genes were exhibited on the SGN database and further confirmed by the NCBI database. The conserved domains of AKR candidate proteins were searched on the Pfam database with the No. of pfam00248. Conserved protein domains were further confirmed by InterPro, which showed that all 28 members contained at least one AKR domain. As a result, these members were considered the candidate genes of the *AKR* family. The total messages of the *SlAKR* family, including genomic location, coding sequence length (CDS), exon number, protein length, protein molecular weight (MW), and isoelectric point (PI), are listed in Table 1 and labeled using their gene loc no. on SGN. *Solyc09g074310.3.1* had the most exons, up to 12, with a CDS length of 1080 bp encoding 359 amino acids but was not the longest gene in the *SlAKR* family. *Solyc11g067160.2.1* was the longest gene with 1197 bp CDS encoding 398 amino acids with nine exons, and the other members of the *SlARK* family had exons ranging from 2 to 10. The shortest gene was *Solyc09g097990.1.1,* with 273 nucleotides encoding only 90 amino acids. The MW of SlARK family proteins ranged from 10,277.02 D to 44,437.18 D, and PI was bridged from 5.12 to 9.28.

### 2.2. Locations of SlAKR on the S. lycopersicum Genome

**Figure 1:**
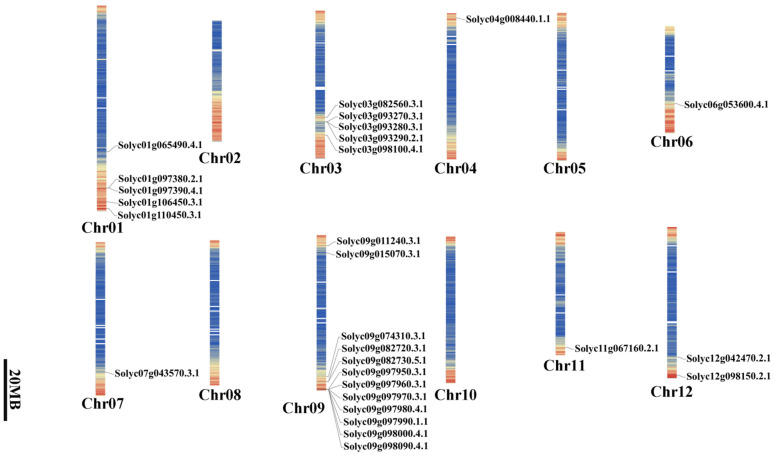
Genomic locations of the *SlAKR* gene on the genome of *Solanum lycopersicum*.

Two chromosomes were found in *S. lycopersicum*, but 28 *SlAKR* family genes were unevenly distributed on eight chromosomes, i.e., Chr.01, Chr.03, Chr.04, Chr.06, Chr.07, Chr.09, Chr.11, and Chr.12 (Figure 1). Most genes were located on Chr.09 with 12 members, and five genes each were found on Chr.01 and Chr.05 (Figure 1). However, one gene each was observed on Chr.04, Chr.06, and Chr.07. Four members of the *AtAKR* family were found in the Tair database, with three genes located on Chr.2 and only one gene located on Chr.3 (Table S1). However, 22 *AKR* family genes were found in rice and unevenly distributed on 10 of 12 chromosomes in rice (Table S1). These results showed that the *AKR* genes of *S. lycopersicum* were 7- and 1.5-fold more than those of *A. thaliana* and rice, respectively. Moreover, the *AKR* genes of rice were distributed more asymmetrically than those of tomato.

### 2.3. Phylogenic Relationship and Gene Structures of AKR Genes in S. lycopersicum

**Figure 2:**
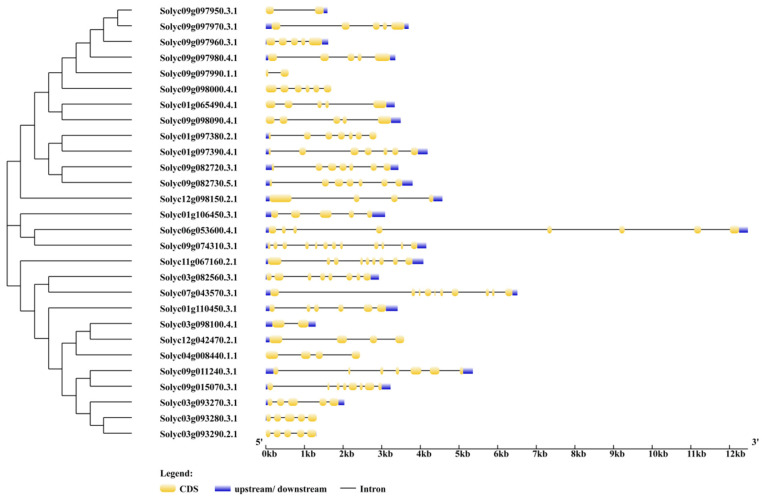
Phylogenic relationship of CDS and gene structures of *AKR* genes in *Solanum lycopersicum*. Phylogenic tree of *SlAKR* CDS was built by the neighbor-joining method, and the gene structures were analyzed by GSDS2.0 according to both genome and CDS sequences.

The CDS sequences of *SlAKRs* were downloaded from the SNG database as fasta forms and aligned by MEGA11 to build the phylogenic tree by the neighbor-joining method. All of the 28 *SlAKR* genes were clustered into three subfamilies, i.e., I, II, and III. Subfamily I was the largest subfamily with 13 members, but the smallest subgroup, i.e., subfamily II, only had three members. Furthermore, the gene structures of *SlAKR* genes were analyzed in online tools (Figure 2). The *Solyc09g074310.3.1* of subfamily II had the most exons, up to 12, but the two other members had 5 and 8 exons. However, the *SlAKR* genes of subfamily I had fewer exons ranging from 2 to 7 than subfamily II with 5 to 10 exons.

### 2.4. Alignment and Phylogenic Analysis of the AKR Protein in S. lycopersicum

**Figure 3:**
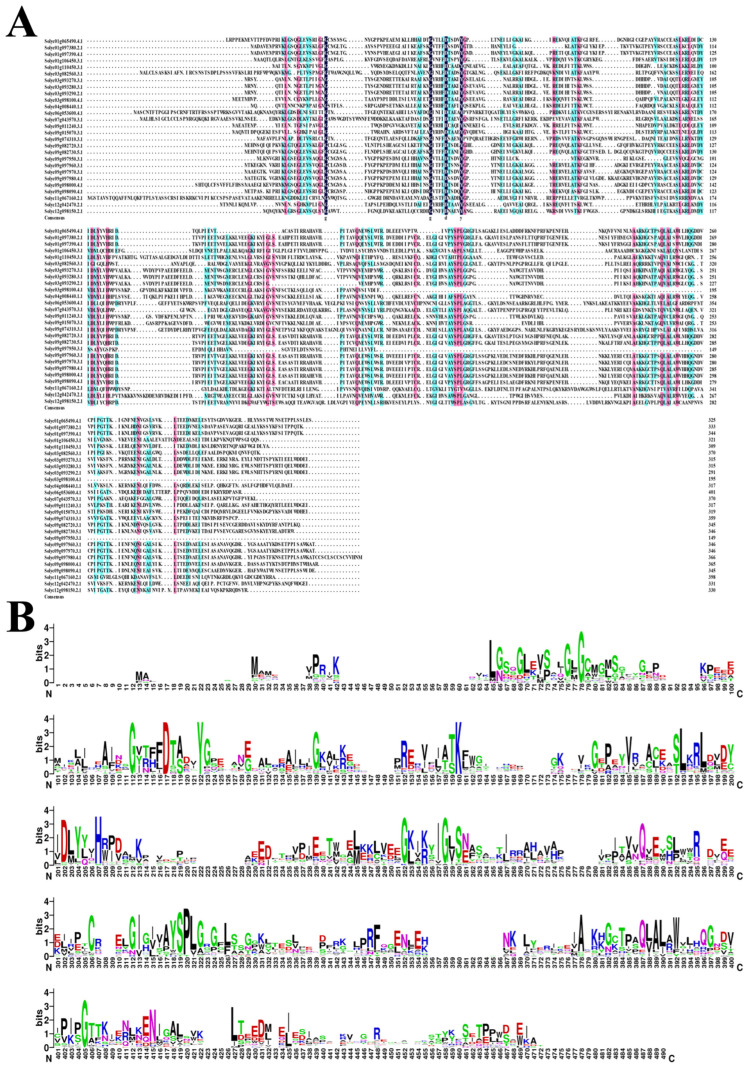
Multiple protein sequence alignments of the SlAKR family in *Solanum lycopersicum*. (**A**) Multiple sequence alignment of SlAKRs. Dark blue represented homology level of 100%, ≥75%, and ≥50% with the colors of blue and, pink, respectively; (**B**) Sequence LOGO of AKRs in *Solanum lycopersicum* on the basis of protein sequences.

First, all SlAKR protein sequences were downloaded from the SGN database and aligned by the DNAMAN8 software (Figure 3A). On the basis of the multiple alignments of the SlAKR protein, four critical conserved sites were found in the front of these sequences labeled as g, g, d, and y in Figure 3A. These sequences were further subjected to cluster analysis by WEBLOGO [22], as shown by the increase in other conserved sites in Figure 3B. Furthermore, the conserved domains of AKR existed in SlAKRs. The positions of each conserved domain is listed in Table S2.

**Figure 4:**
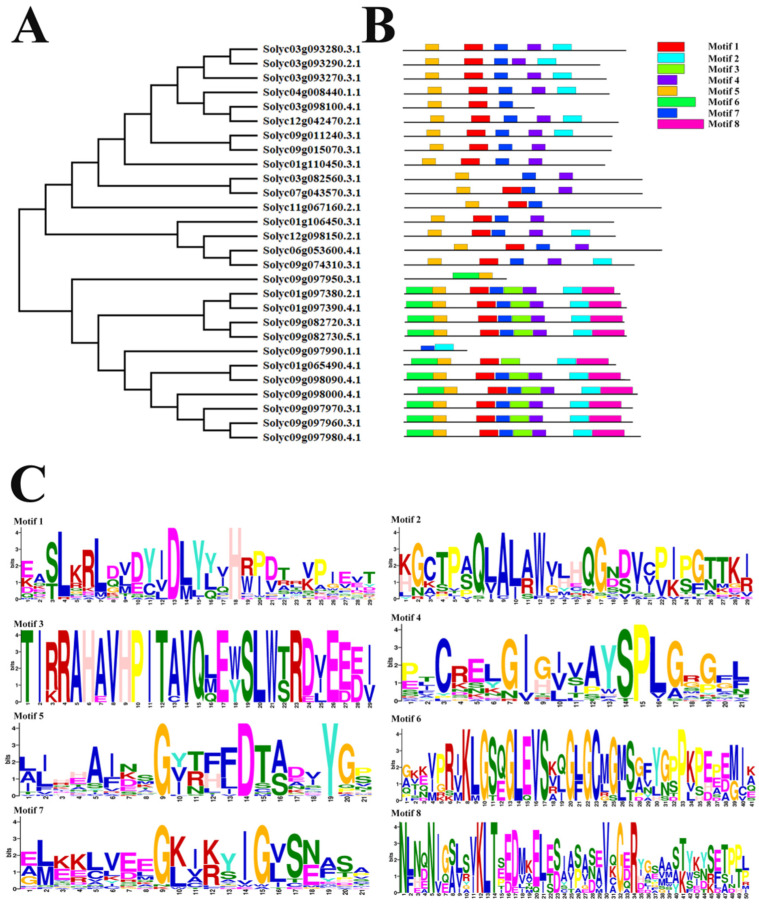
Phylogenic tree of protein and motifs for the SlAKR family. (**A**) Phylogenic tree of SlAKR protein by the neighbor-joining method in MEGA11 with a bootstrap of 1000; (**B**) Motif analysis of each SlAKR protein by MEME. Differently colored boxes represent different motifs; (**C**) The amino acid sequence logo of each motif in SlAKR proteins. The size of the letters represents the frequency of occurrence.

The phylogenetic relationships of 28 SlAKR proteins were calculated by the neighbor-joining method in MEGA11 with a bootstrap of 1000, Figure 4A. The protein motifs of SlAKRs were searched by the online tool MEME and distributed on each SlAKR with 8 motifs in Figure 4B. Among these motifs, the most conservative motif 5 existed in 27 members of SlAKR, and motif 7 existed in 26 proteins. Following Motif 1 existed in 25 members except for Solyc03g082560.3.1, Solyc09g097950.3.1 and Solyc09g097990.1.1. The WEBlogo analysis results of each motif in SlAKR proteins are shown in Figure 4C and Table S3. Three loops, i.e., Loops A, B, and C, in the AKR domain were reported. Our results showed that motif 1 comprised the most conserved structures of AKR, such as Loop A.

### 2.5. Phylogenic Relationships of AKR Protein in S. lycopersicum, A. thaliana, and Oryza sativa

**Figure 5:**
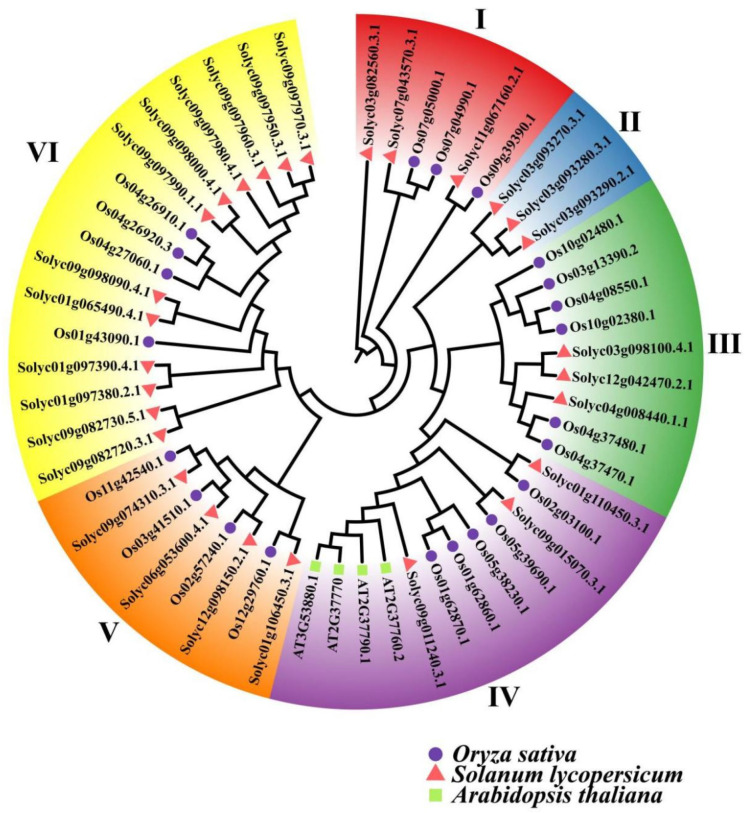
Phylogenic relationship of the AKR protein family in *Solanum lycopersicum*, *Arabidopsis thaliana*, and *Oryza sativa.* Red triangles represent *Solanum lycopersicum*, green squares represent *Arabidopsis thaliana*, and purple circles represent *Oryza sativa*.

The protein sequences of 28 SlAKRs, 22 OsAKRs, and 4 AtAKRs (Table S4) were obtained from the database to construct the circular evolutionary tree by the neighbor-joining method to explore the phylogenic relationships of the AKR protein among *S. lycopersicum*, *A. thaliana*, and *O. sativa*. All AKR proteins of these three species were divided into six subgroups, i.e., I to VI, labeled with different colors (Figure 5). All four AtAKRs were clustered into a sub-branch in subgroup IV. However, the AKRs of rice were distributed in many more subfamilies with similarities to *S. lycopersicum* in each subgroup. Subgroup II only had three AKR members.

### 2.6. Collinearity Relationship of AKR Genes in S. lycopersicum, A. thaliana, and O. sativa

**Figure 6:**
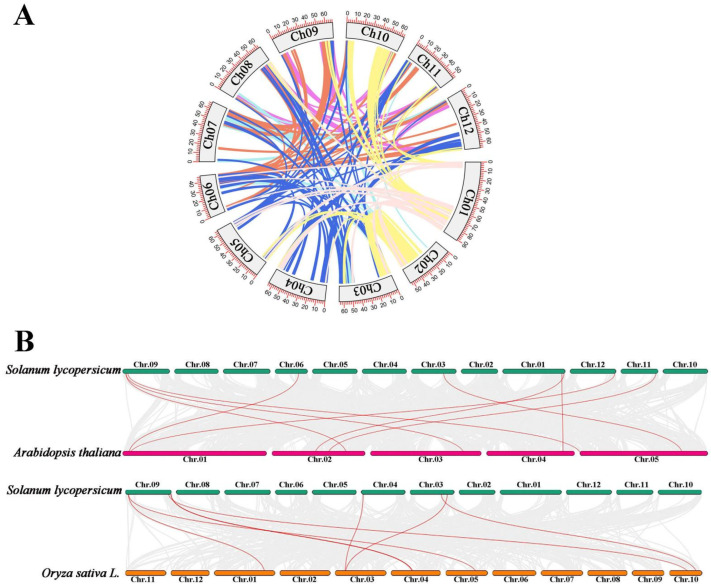
Collinearity relations of *AKR* in *Solanum lycopersicum*, *Arabidopsis thaliana,* and *Oryza sativa*. (**A**) Collinearity analysis of *SlAKR* family genes in *Solanum lycopersicum*; (**B**) Collinearity analysis of *AKR* family genes not only between *Solanum lycopersicum* and *Arabidopsis thaliana* but also between *Solanum lycopersicum* and *Oryza sativa*.

The collinearity analysis of *AKR* genes was carried out (Figure 6) to study the repetition events of the *AKR* gene family in *S. lycopersicum*, and the distribution and arrangement relationships of *AKR* genes between *S. lycopersicum* and other species were explored. First, the synteny of *SlAKR* genes was analyzed (Figure 6A). A total of 34 075 genes were found in the genome of *S. lycopersicum* with the Version SL4.0, among which 7217 genes were collinearity genes accounting for 21.18%. However, no collinearity gene was observed among the *SlAKR* gene family. Second, the collinearity relationships of *AKRs* among the dicot plant *A. thaliana*, monocot plant *O. sativa*, and *S. lycopersicum* were determined (Figure 6B). Nine pairs of collinearity genes, i.e., *Solyc06g053600.4.1* and *AT1G04420.1*, *Solyc12g098150.2.1* and *AT1G04690.1*, *Solyc01g110450.3.1* and *AT2G21250.1*, *Solyc09g011240.3.1* and *AT2G37760.1, Solyc11g067160.2.1* and *AT2G27680.1, Solyc09g011240.3.1* and *AT3G53880.1, Solyc01g106450.3.1* and *AT4G33670.1, Solyc03g082560.3.1* and *AT5G53580.1,* and *Solyc09g015070.3.1* and *AT5G01670.2*, were found between *A. thaliana* and tomato. Eight pairs of collinearity genes, i.e., *Solyc09g011240.3.1* and *Os01t0847600-01, Solyc09g082720.3.1* and *Os10t0419100-01, Solyc09g098090.4.1* and *Os04t0337500-01, Solyc09g097990.1.1* and *Os04t0341100-00, Solyc09g011240.3.1* and *Os05t0456300-01, Solyc04g008440.1.1* and *Os03t0237100-01, Solyc03g082560.3.1* and *Os10t0517400-01,* and *Solyc03g098100.4.1* and *Os03t0237100-01* were found between rice and tomato. Interestingly, *Solyc09g011240.3.1* had collinearity genes in both *A. thaliana* and rice, such as *AT3G53880.1* and *Os01t0847600-01*, *Os05t0456300-01*, but no collinearity genes of tomato, indicating that *Solyc09g011240.3.1* had gene sequence repetition events in rice and *Arabidopsis* but not in tomato.

### 2.7. Expression Levels of SlAKR Family Genes in Various Tissues

**Figure 7:**
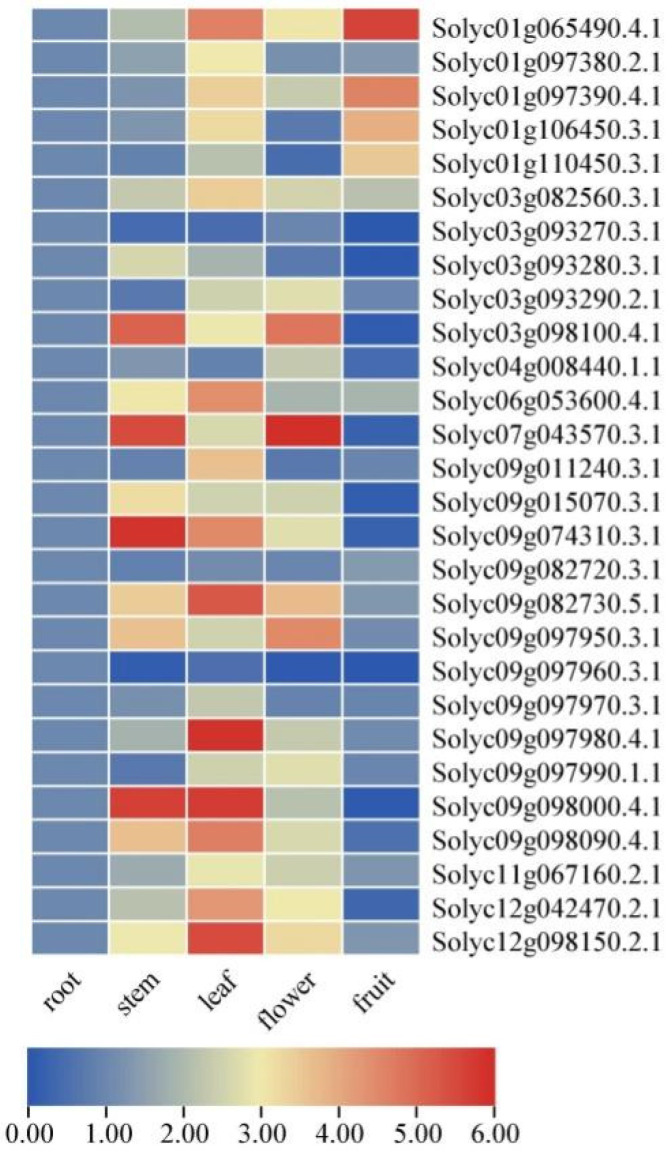
The expression levels of *SlAKR* genes in various organs of *Solanum lycopersicum*.

The transcriptional levels of *SlAKR* family genes were detected by qPCR in different organs and tissues, including roots, stems, leaves, flowers, and fruits with a diameter of 1 cm, to investigate the potential functions of *SlAKRs* in tomatoes (Figure 7). *SlAKR* genes widely enhanced their expression levels in stems, leaves, and flowers and leaves. Compared with roots, the expression levels of most *SlAKRs* (85.7%) were significantly increased in leaves, such as *Solyc09g097980.4.1,* with the highest expression. The transcriptional levels of the majority of *SlAKRs* genes were downregulated in fruits, but four genes, such as *Solyc01g065490.4.1*, *Solyc01g097390.2.1*, *Solyc01g106450.3.1,* and *Solyc01g100450.3.1,* in fruit were upregulated than those in other tissues. However, some *SlAKRs,* such as *Solyc03g093270.3.1* and *Solyc09g097960.3.1,* had low expression levels in all tissues.

### 2.8. Expression Profiles of SlAKR Family Genes under Drought and Salt Stresses in AC Lines

**Figure 8:**
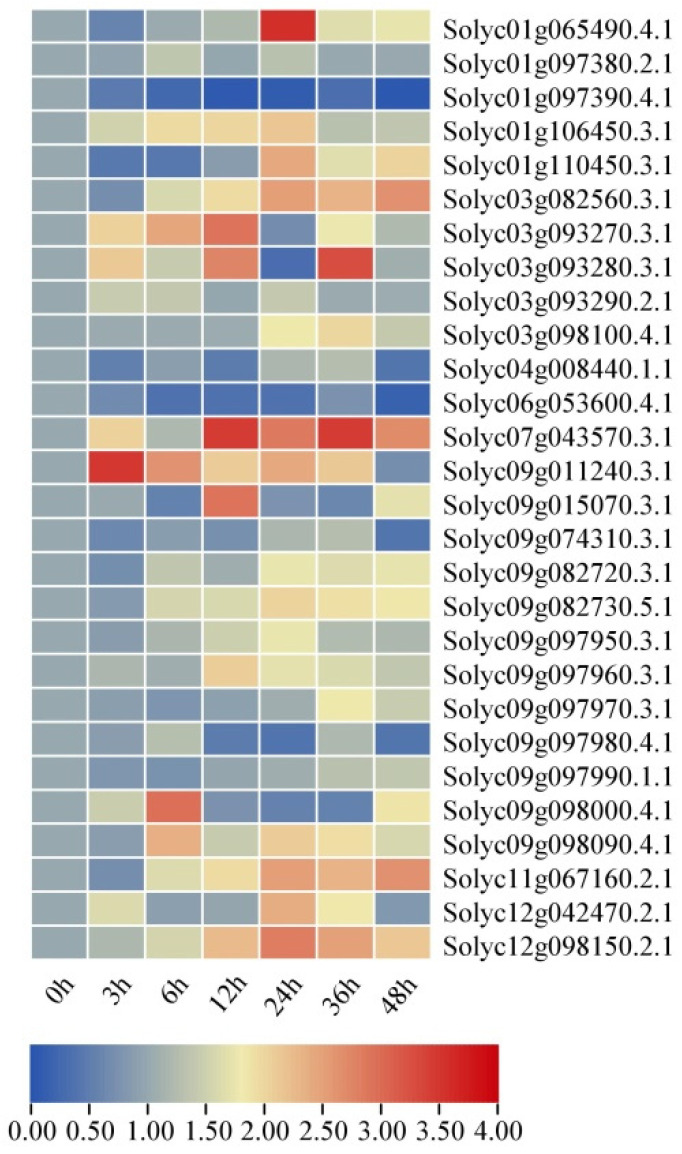
The expression levels of *SlAKR* genes in response to drought stresses treated with 10% PEG for different times, such as 0, 3, 6, 12, 24, 36, and 48 h, with dd H_2_O as a control in AC.

**Figure 9:**
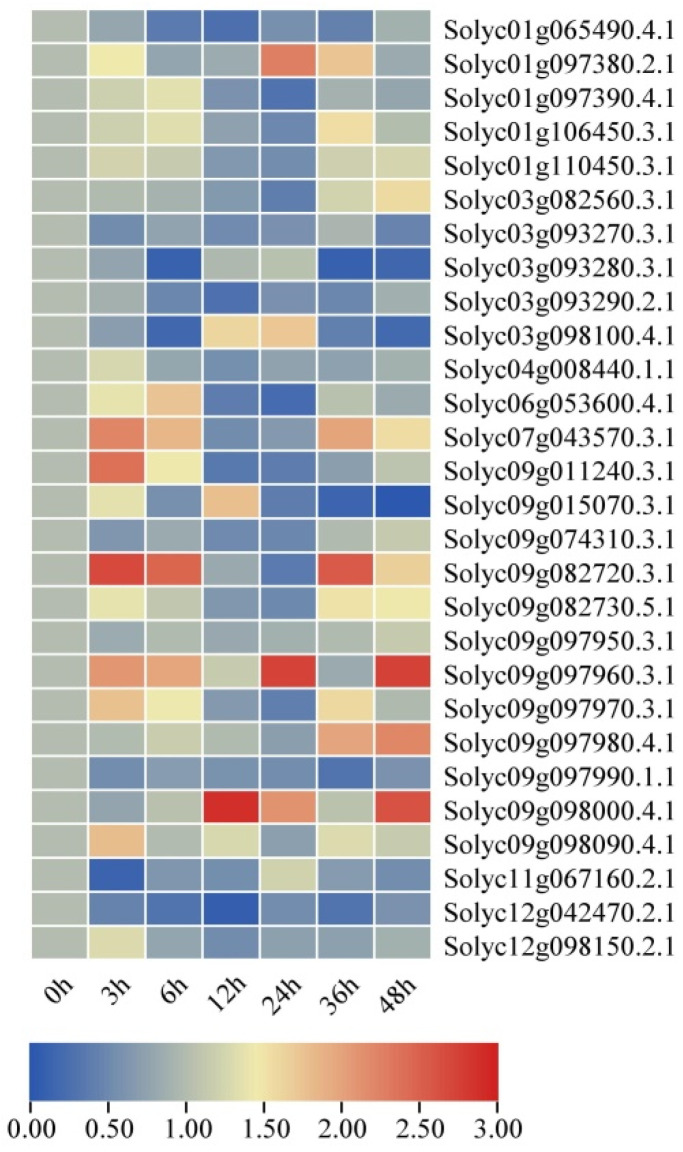
The expression levels of *SlAKR* genes in response to salt stresses treated with 200 mM NaCl for different times, such as 0, 3, 6, 12, 24, 36, and 48 h, with dd H_2_O as a control in AC.

The tomato AC inbred lines were treated with 10% PEG6000 as drought conditions and 200 mM NaCl as salt stresses for 0, 3, 6, 12, 24, 36, and 48 h to further explore the functions of *SlAKRs* in response to abiotic stresses. All plants were harvested for the expression profiles of the *SlAKRs* family by real-time fluorescent quantitative PCR (Figure 8 and Figure 9). In response to drought stresses, the expression levels of most *SlAKR* genes (75%) were significantly induced at 24 h treatment of 10% PEG6000 treatments and then decreased at 48 h of drought treatments. *Solyc03g082560.3.1*, *Solyc07g043570.3.1*, *Solyc11g067160.2.1*, and *Solyc12g098150.2.1* enhanced their expressions at all time points of drought, but some *SlAKR* genes, such as *Solyc01g097390.4.1* and *Solyc06g053600.4.1,* maintained low expression levels at each treatment time.

Under salt stresses, the expression patterns of *SlAKRs* became diversified. The expression levels of the majority of *SlAKR* genes were upregulated at 3 h salt stresses, which showed more rapid responses than drought stress. However, the expression patterns of *SlAKRs* except *Solyc01g097380.2.1*, *Solyc09g098000.4.1,* and *Solyc09g097960.3.1* decreased substantially for salt stresses at 12 and 24 h. A large amount of increased expressions occurred at 36 and 48 h, such as *Solyc07g043570.3.1*. Furthermore, only one gene was downregulated in all treatment time points. These results showed that salt stresses induced fewer *SlAKR* genes than drought treatments, which revealed that *SlAKR* genes were dramatically responsive to drought stresses.

### 2.9. Functional Analysis of Silencing Solyc09g0112403.1, Solyc07g043570.3.1, and Solyc01g106450.3.1 in S. lycopersicum

*Solyc09g011240.3.1, Solyc07g043570.3.1,* and *Solyc01g106450.3.1* were significantly changed in response to drought and salt stresses, which indicated that they might play roles in abiotic stresses. About 400–600 bp of these three genes were cloned and constructed to the pTRV2 vector derived by the *35S*-mediating virus-induced gene silencing (VIGS) system. The recombinant plasmids of *35S*:pTRV2-*Solyc09g011240.3.1*, *35S*:pTRV2-*Solyc07g043570.3.1*, and *35S*:pTRV2-*Solyc01g106450.3.1* were transformed into tomato seedlings via *Agrobacterium*-mediated transformation. The pTRV2 vector and pTRV2- phytoene desaturase (*PDS*) were also transformed into the tomato, which seemed indicators. Recombinant plasmids were transformed into 50 independent tomato seedlings, and the expression levels of each target gene were detected by qPCR (Table S5) to calculate the silencing efficiency when the silenced transgenic tomatoes of *PDS* turned white. The silencing efficiency of these three target genes was up to 40–70% (Figure S1), and 15 transgenic tomato lines with similar silencing efficiency were selected for the next detection of physiological indicators.

Malonaldehyde (MDA) content, proline (Pro) content, and peroxidase (POD) enzyme activity in plants are important physiological indices to evaluate the degree of plant resistance to abiotic stresses. MDA is one of the most important products after the lipid peroxidation of the membrane, aggravating membrane damage. POD enzyme is one of the key enzyme defense systems in plants and can eliminate excessive free radicals under adverse conditions to improve the stress resistance of plants. Proline can maintain osmotic balance at the cellular level and is an important osmotic regulator [23]. The silenced transgenic tomatoes of pTRV2:*Solyc09g011240.3.1*, pTRV2:*Solyc07g043570.3.1*, and pTRV2:*Solyc01g106450.3.1* were harvested after treatments of 10% PEG or 200 mM NaCl for 0, 12, 24, 36, and 48 h, and their contents of MDA, Pro, and POD were determined. All silenced transgenic tomato lines had higher MDA content than pTRV2:00 under each treated time point and lower Pro and POD contents than control plants. Among these physiological indices, the silenced transgenic tomatoes of pTRV2:*Solyc09g011240.3.1* were changed most remarkably in the other genes during drought stresses. Under salt stresses, the contents of MDA, POD, and Pro in the silenced transgenic tomatoes of pTRV2:*solyc09g011240.3.1*, pTRV2:*Solyc07g043570.3.1*, and pTRV2:*Solyc01g106450.3.1* were similar to drought stresses. The Pro contents of the silenced plants were lower than those of control plants within 0–24 h after salt treatments but increased at 36 h of salt treatments sharply. Similar to the results of drought stresses, the contents of MDA, POD, and Pro in the silenced transgenic tomatoes of pTRV2:*Solyc09g011240.3.1* were the most distinct compared with those in other silenced transgenic lines. These results revealed that the silenced transgenic tomatoes of *Solyc09g011240.3.1*, *Solyc07g043570.3.1*, and *Solyc01g106450.3.1* were all sensitive to drought and salt stresses compared with pTRV2 controls.

## 3. Discussion

AKRs encoding NADP(H)-dependent oxidoreductases exist in nearly all phyla and function in the phase 1 metabolism of endogenous substrates and xenobiotics [2,4]. More than 190 annotated proteins of *AKRs* were searched by the alignments of genome data classified into 16 families [13,24]. The *AKRs* of humans play multiple roles in a variety of disease processes, and selective enzyme inhibitors have been sought as chemical probes and as possible therapeutics [2]. Plant *AKRs* significantly participate in multiple stresses to confer tolerance, including abiotic responses (such as salt and drought stresses) and biotic stresses (such as pathogen defense) [25]. Moreover, *AKRs* function in primary and secondary metabolic pathways during the growth and development of the plant. With the continuous study of functions, increasing plant *AKR* families, such as *A. thaliana* [10], rice [11], *H. vulgare* [12], and *M. truncatula* [13], have been identified and excavated. However, the genome-wide analysis of the *AKR* gene family has not been performed in tomatoes yet. In our study, 28 *SlAKR* genes were identified from the tomato genome, and the bioinformation and expression levels under abiotic stresses were further analyzed to provide comprehensive information about the *AKR* family in tomatoes.

### 3.1. Analysis of SlAKRs Characterization and Phylogenetic Relationships

All gene structures of 28 *SlAKR* genes were analyzed, and results showed that similar gene structures were clustered in the same subfamily in accordance with the phylogenetic relationships of *SlAKR* genes (Figure 2). Multiple protein sequences, motifs, and conserved domains of *SlAKRs* were compared. The locations of each *AKR* conserved domain were similar in the whole protein, thus contributing to the classical (α/β) 8-barrel fold structures [4]. Moreover, the cluster analysis of *AKR* conserved domains of tomatoes found six highly conserved amino acid sites, such as glycine (G-78), glycine (G-112), aspartic acid (D-117), tyrosine (Y-122), lysine (K-160), and glycine (G-405) in Figure 3B, which were the conserved sites of most *AKRs* in 40 different plant species [25]. The 28 SlAKRs proteins encoded an average of 318 amino acids with MW of 35.6 KD, which was similar to the AKRs reported previously about 320 amino acids with 33–37 kD [2]. However, MtAKRs contained 336 amino acids on average, with 37 kDa in *M. truncatula* [13], and this value was slightly larger than average AKRs and even SlAKRs. Only one conservative motif was found in all 28 SlAKRs encoding the AKR domain.

*SlAKRs* were not evenly distributed on 12 chromosomes, as shown in Figure 1. No *SlAKR* gene was located on chromosomes 2, 5, 8, and 10. Most *SlAKR* genes(12) on chromosome 9 were not even either. The uneven distribution of genes may be related to species evolution and genetic variation [26]. No collinearity gene was observed among the *SlAKR* gene family (Figure 6A), and no duplication event of *SlAKR* was observed on the genome. However, 9 and 8 pairs of collinearity genes were found among tomato, *A. thaliana,* and rice, indicating that *SlAKR* genes shared similar syntenic relationships with *AtAKRs* and *OsAKRs*. These collinearity genes shared close phylogenetic relationships in different species, but *Solyc09g011240.3.1* had collinearity genes in *A. thaliana* and rice.

### 3.2. Expression Profiles and Candidate Genes of SlAKRs

**Figure 10:**
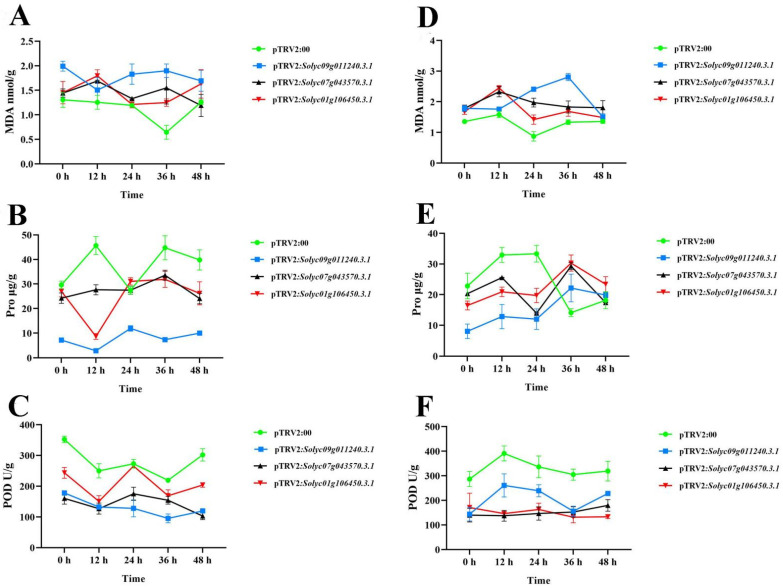
Physiological indicators of silenced transgenic tomato lines under drought and salt stresses for 0, 3, 6, 12, 24, 36, and 48 h with AC as control. The MDA contents (**A**), the Pro activities (**B**), and the POD activities (**C**) of transgenic tomato lines, including pTRV2:*Solyc09g011240.3.1*, pTRV2:*Solyc07g043570.3.1*, and pTRV2:*Solyc01g106450.3.1* under 10% PEG stresses for 0, 3, 6, 12, 24, 36, 48 h with AC as control. The MDA contents (**D**), the Pro activities (**E**), the POD activities (**F**) of transgenic tomato lines, including pTRV2:*Solyc09g0112403.1*, pTRV2:*Solyc07g043570.3.1,* and pTRV2:*Solyc01g106450.3.1* under 200 mM NaCl stresses for 0, 3, 6, 12, 24, 36, and 48 h, with AC as a control.

The expression profiles of 28 *SlAKRs* in different tissues, such as root, stem, leaves, flower, and fruit, were detected by qPCR, which showed that most *SlAKRs* were induced in stem, leaves, and flower. *SlAKRs* had low expression profiles in root and fruit. *Solyc03g093270.3.1*, *Solyc09g082720.3.1*, and *Solyc09g097960.3.1* had low expression levels in all tissues. *Solyc09g074310.3.1* and *Solyc09g097980.4.1* were specifically expressed in stems and leaves, respectively, which indicated that the tissue-specific expression of the *SlAKR* family members might be related to biological functions. *SlAKRs* consist of *MtAKRs* [13]. Plant *AKRs* play multiple roles in abiotic and biotic stress defenses, including ozone, drought, salinity, hypoxia, and *P. syringae* inoculation [18,27,28,29]. *MsALR*, *AKR4C8,* and *AKR4C9* are reported to be highly induced under abiotic stress [10,30] because *AKR* mediates stress tolerance by detoxification and ROS clearance in cell-damaging reactions [25]. Moreover, the overexpression of barley *AKR4C9* enhances their tolerance in response to freezing, oxidative, and cadmium stresses [8,31]. Thus, the expression profiles of *SlAKR* genes in response to drought and salt stresses were detected in each treatment. Most *SlAKR* genes were induced by drought treatments at various times. Among these genes, *Solyc01g065490.4.1, Solyc07g043570.3.1*, and *Solyc09g011240.3.1* showed significant induction. However, results due to salt stresses were less dramatically changed compared with those due to drought. *Solyc07g043570.3.1, Solyc09g011240.3.1, Solyc09g082720.3.1*, and *Solyc09g097960.3.1* showed rapid increases in their expression levels in response to salt stresses. On the basis of expression characteristics, *Solyc09g011240.3.1*, *Solyc07g043570.3.1*, and *Solyc01g106450.3.1* were silenced in tomatoes by the VIGS system, which showed sensitivity to drought and salt stresses with low contents of Pro and POD and high content of MDA at each stress stage (Figure 10). The overexpression of *OsAKR4C15* in *A. thaliana* lines resulted in lower contents of MDA and MG under both stresses and control conditions than wild types [32]. These results suggested that *AKR* members of tomatoes were in response to drought and salt stresses and that *Solyc09g011240.3.1*, *Solyc07g043570.3.1*, and *Solyc01g106450.3.1* might play positive roles in regulating the tolerance of tomatoes in drought and salt stresses.

## 4. Materials and Methods

### 4.1. Plant Materials

Tomato Ailsa Craig (AC) were sown in the soil and transferred to a greenhouse with 16 h/8 h for day/night and temperatures of 25 °C/20 °C until four leaves. A total of 21 seedlings of tomatoes were treated with 200 mM NaCl for 0, 3, 6, 12, 24, 36, and 48 h with water as control, and 21 other seedlings were treated with 10% PEG 6000 for the same treated time as NaCl treatment with three biological replicates. A total of 126 tomato seedlings were harvested, frozen quickly in liquid nitrogen, and stored at −80 °C.

When AC tomato seedlings grew to the flowering stage, different tissues of three independent seedlings, including roots, stems, leaves, flowers, and 1 cm green fruits, were collected. All parts of the tissue were frozen with liquid nitrogen and stored at −80 °C.

### 4.2. RNA Extraction and Real-Time PCR

The plant samples of three biological replicates were ground into powder in liquid nitrogen, and the total RNA was isolated by the Trizol reagent (Invitrogen, Thermo Fisher Technology Co., LTD., Waltham, MA, USA) in accordance with the manufacturer’s manual and reversely transcribed into cDNA by the Vazyme reverse transcription kit (Vazyme Code: R223-01, Vazyme Biotech Co., Ltd., Nanjing, China).

The total volume of qPCR was 15 μL:7.5 μL qPCR-Mix (Vazyme Biotech Co., Ltd.). The cDNA was diluted 20 times, and 3 μL was collected as a template. About 0.5 μL forward and reverse primers were added with dd H_2_O to obtain a volume of 3.5 μL. The PCR system was subjected to abi-q3 fluorescence quantitative PCR with a three-step procedure with three biological replicates [33]. The PCR primers of *SlAKRs* were designed by Premier Express software Ver in the nonconservative areas of each *SlAKR* (Table S5), and all genes were normalized by the reference gene *SlActin*.

### 4.3. Isolation of AKR Family Members

AKR family members were searched on the SGN (https://solgenomics.net/ (accessed on 12 July 2021)) and NCBI (https://www.ncbi.nlm.nih.gov/ (accessed on 20 July 2021)) databases, and candidate genes with the conserved domains of AKR, such as pfam00248, were screened out. The conserved domains of the AKR family were searched on the Pfam (http://pfam.xfam.org/ (accessed on 10 August 2021)). All CDS, genome, and protein sequences of AKR were downloaded in FASTA format from the SGN database. All proteins of AKR were confirmed by InterPro (http://www.ebi.ac.uk/interpro/ (accessed on 10 August 2021)). The MW and PI values of tomato AKR members were analyzed by the online ExPASy website (https://web.expasy.org/compute_pi/ (accessed on 10 August 2021)).

### 4.4. Location and Structures of ARK Members

The location of *SlAKR* members and gene distribution of tomato chromosomes were obtained from the annotations on SGN (https://solgenomics.net (accessed on 20 July 2021)), which were redrawn by TBtools [34]. The gene structures of *SlAKR* were analyzed by an online tool GSDS2.0 (http://gsds.gao-lab.org/ (accessed on 15 August 2021)) [35] in accordance with genome and CDS sequences.

### 4.5. Phylogenetic Analysis

The AKR protein sequences of *A. thaliana* and rice were downloaded from the Tair (https://www.arabidopsis.org (accessed on 25 July 2021)) and Gramene (https://www.gramene.org/ (accessed on 25 July 2021)) databases. The protein sequences of *A. thaliana*, rice, and tomatoes and the CDS sequences of tomatoes were blasted by MEGA 11, and phylogenetic trees were built using the online tool iTOL: Interactive Tree of Life (https://itol.embl.de/upload.cgi (accessed on 15 January 2022)) through the neighbor-joining method. The motifs of the tomato AKR family were searched on the MEME database (http://meme-suite.org/tools/meme (accessed on 21 March 2022)).

### 4.6. Synteny

The distributions and interchromosomal relationships of *SlAKRs* were obtained by MCScanX associated with TBtools. The synteny relationships of *AKRs* family members between *A. thaliana* and rice were also determined by MCScanX [36] and TBtools [34]. The genome and coding sequences of *AKR* in *A. thaliana* and rice were obtained from Tair and Gramene databases.

### 4.7. Construction of Recombinant Plasmids and Transformation of Tomato

Three candidate genes of the *AKR* family were amplified for 300–500 bp fragments to be constructed to the VIGS vectors pTRV2 with specific primers (Table S6), such as *Solyc01g106450.3.1*, *Solyc07g043570.3.1*, and *Solyc09g011240.3.1*. Recombinant plasmids were screened, sequenced, and then transformed into tomato AC by *Agrobacterium tumefaciens* (EHA105) injection with pTRV2 as the negative control. The albino gene of *PDS* was also transferred in AS tomatoes as the display agent.

### 4.8. Measurement of Physiological Indices

The transgenic tomatoes of pTRV2-*Solyc01g106450.3.1*, pTRV2-*Solyc07g043570.3.1*, and pTRV2-*Solyc09g11240.3.1* were detected by qPCR with pTRV2 as control when the transgenic tomatoes of PDS turned to white. A total of 21 transgenic tomato seedlings were treated with 200 mM NaCl or 10% PEG 6000 at different times and harvested for measurements of Pro, POD, and MDA contents by using the proline (PRO-1-Y, Suzhou Comin biotechnology Co., Ltd., Suzhou, China), POD (POD-1-Y, Suzhou Comin Biotechnology Co., Ltd., Suzhou, China) and MDA (MDA-1-Y, Suzhou Comin Biotechnology Co., Ltd., Suzhou, China) content kits, respectively. Analysis was carried out with three biological replicates.

## Figures and Tables

**Table 1:** The *AKR* family genes in *Solanum lycopersicum*.

Gene ID	Location	Exon	CDS (bp)	Protein (aa)	MW (da)	PI
Solyc01g065490.4.1	Ch01:64503126..64507794	5	961	319	35,408.56	5.46
Solyc01g097380.2.1	Ch01:80521659..80524521	7	1002	333	36,601.78	5.47
Solyc01g097390.4.1	Ch01:80526571..80530758	7	1036	344	38,093.9	7.14
Solyc01g106450.3.1	Ch01:86648540..86651626	5	967	321	34,659.69	5.4
Solyc01g110450.3.1	Ch01:89495104..89498514	6	931	309	34,691.84	6.15
Solyc03g082560.3.1	Ch03:47065999..47068924	8	1113	370	44,692.65	9.28
Solyc03g093270.3.1	Ch03:49011703..49013733	5	947	315	35,907.79	5.12
Solyc03g093280.3.1	Ch03:49018029..49019346	5	948	315	36,067.09	5.75
Solyc03g093290.2.1	Ch03:49025805..49027630	5	876	291	33,388.95	5.75
Solyc03g098100.4.1	Ch03:54950796..54952085	2	589	195	21,617.42	5.81
Solyc04g008440.1.1	Ch04:2097095..2100505	4	954	317	35,736.61	7.11
Solyc06g053600.4.1	Ch06:34195838..34208314	8	1207	401	45,400.67	7.59
Solyc07g043570.3.1	Ch07:57339225..57345735	10	1069	355	39,163.51	7.65
Solyc09g011240.3.1	Ch09:4605903..4611260	7	955	317	35,568.88	6.27
Solyc09g015070.3.1	Ch09:7921034..7924262	8	961	319	36,210.64	5.97
Solyc09g074310.3.1	Ch09:62302505..62306658	12	1080	359	40,259.85	6.20
Solyc09g082720.3.1	Ch09:64521009..64524440	7	1039	345	38,306.89	5.91
Solyc09g082730.5.1	Ch09:64525521..64529319	7	1042	346	38,202.57	5.43
Solyc09g097950.3.1	Ch09:67972899..67974512	5	450	149	16,033.51	6.03
Solyc09g097960.3.1	Ch09:67972899..67974512	5	1042	349	38,212.53	5.87
Solyc09g097970.3.1	Ch09:67974976..67978670	5	1042	346	37,766.09	5.9
Solyc09g097980.4.1	Ch09:67978934..67982284	5	1057	351	38,574.17	6.24
Solyc09g097990.1.1	Ch09:67983109..67983935	2	273	90	10,277.02	8.48
Solyc09g098000.4.1	Ch09:67985560..67987253	6	1051	349	38,675.3	5.92
Solyc09g098090.4.1	Ch09:68042656..68046145	5	1038	345	38,729.75	5.61
Solyc11g067160.2.1	Ch11:50922853..50926930	9	1197	398	44,437.18	8.85
Solyc12g042470.2.1	Ch12:57401753..57405333	4	996	331	37,500.62	6.47
Solyc12g098150.2.1	Ch12:65298986..65303554	4	993	330	36,956.2	7.61

## Data Availability

SGN (https://solgenomics.net/), NCBI (https://www.ncbi.nlm.nih.gov/), the Pfam (http://pfam.xfam.org/), InterPro (http://www.ebi.ac.uk/interpro/). ExPASy website (https://web.expasy.org/compute_pi/, GSDS2.0 (http:// gsds.gao-lab.org/), Tair (https://www.arabidopsis.org), Gramene (https://www.gramene.org/), iTOL: Interactive Tree of Life (https://itol.embl.de/upload.cgi), MEME database (http://meme-suite.org/tools/meme).

## Supplementary Materials

The following supporting information can be downloaded at: https://www.mdpi.com/article/10.3390/ijms24021272/s1.
