# Supplementary material for: Genome-Wide Identification and Characterization of Aldo-Keto Reductase (AKR) Gene Family in Response to Abiotic Stresses in Solanum lycopersicum
Source: Int J Mol Sci. 2023 Jan 9;24(2):1272. doi: 10.3390/ijms24021272 (PMC9865680; doi:10.3390/ijms24021272)
Supplement: Supplementary file 1 [file ijms-24-01272-s001.zip › ijms-2080796-supplementary.pdf]

Table S1. The Chromosome locations of *Aldo-Keto Reductases*（*AKR*） in *Arabidopsis thaliana, Oryza sativa* and *Solanum lycopersicum*

	Chr.1	Chr.2	Chr.3	Chr.4	Chr.5	Chr.6	Chr.7	Chr.8	Chr.9	Chr.10	Chr.11	Chr.12
Arabidopsis thaliana	0	3	1	0	0	-	-	-	-	-	-	-
Oryza sativa	3	2	2	6	2	0	2	0	1	2	1	1
Solanum lycopersicum	5	0	5	1	0	1	1	0	12	0	1	2

Table S2 The positions of conserved domain in SlAKRs

GeneID	Domain name	Position(aa)
Solyc01g065490.4.1	Aldo_ket_red	10-285
Solyc01g097380.2.1	Aldo_ket_red	8-300
Solyc01g097390.4.1	Aldo_ket_red	8-311
Solyc01g106450.3.1	Aldo_ket_red	21-306
Solyc01g110450.3.1	Aldo_ket_red	14-291
Solyc03g082560.3.1	Aldo_ket_red	55-352
Solyc03g093270.3.1	Aldo_ket_red	17-269
Solyc03g093280.3.1	Aldo_ket_red	17-281
Solyc03g093290.2.1	Aldo_ket_red	17-257
Solyc03g098100.4.1	Aldo_ket_red	21-190
Solyc04g008440.1.1	Aldo_ket_red	16-292
Solyc06g053600.4.1	Aldo_ket_red	60-391
Solyc07g043570.3.1	Aldo_ket_red	39-341
Solyc09g011240.3.1	Aldo_ket_red	21-287
Solyc09g015070.3.1	Aldo_ket_red	27-284
Solyc09g074310.3.1	Aldo_ket_red	19-350
Solyc09g082720.3.1	Aldo_ket_red	24-313
Solyc09g082730.5.1	Aldo_ket_red	24-320
Solyc09g097950.3.1	Aldo_ket_red(2)	21-70、96-145
Solyc09g097960.3.1	Aldo_ket_red	24-315
Solyc09g097970.3.1	Aldo_ket_red	24-315
Solyc09g097980.4.1	Aldo_ket_red	24-320
Solyc09g097990.1.1	Aldo_ket_red	5-74
Solyc09g098000.4.1	Aldo_ket_red	25-317
Solyc09g098090.4.1	Aldo_ket_red	23-314
Solyc11g067160.2.1	Aldo_ket_red	78-374
Solyc12g042470.2.1	Aldo_ket_red	25-301
Solyc12g098150.2.1	Aldo_ket_red	18-319

Table S3 Motif Sequences

Name	Sequences
Motif 1	EASLKRLDVDYIDLYYVHRPDTRVPIEVT
Motif 2	KGCTPAQLALAWVLHQGBDVCPIPGTTKI
Motif 3	TIRRAHAVHPITAVQLEWSLWTRDVEEEI
Motif 4	PTCRELGIGIVAYSPLGRGFL
Motif 5	LJHEAINSGVTFFDTADVYGP
Motif 6	GKEVPRIKLGSZGLEVSKQGLGCMGMSGFYGPPKPEPEMIK
Motif 7	ELKKLVEEGKIKYIGVSNASA
Motif 8	NLNQNIGALSVKLTEEDMKELESIASASAVKGERYGSAASTYKYSETPPL

Table S4. ***Aldo-Keto Reductases*(*AKR*) genes in *Arabidopsis thaliana*,*Oryza sativa* and *Solanum lycopersicum***

Name	No.
Arabidopsis thaliana	4
Oryza sativa	22
Solanum lycopersicum	28

Table S5 Primers of qPCR

Gene No.	Forward primer Sequence (5'-3')	Reverse primer Sequence (5'-3')
Solyc01g065490.4.1	AGCTGATCGAAAGCTTGGCT	CGTAGTGCCTGGTATAGGGC
Solyc01g097380.2.1	TTTGGCTGTATGGGGCTCAC	TGCAAGCTTCCCGATCAAGT
Solyc01g097390.4.1	GCTCACCCGGAGACGATAAG	TTTCCAGCAAAAAGCCCACG
Solyc01g106450.3.1	AGCAAGCTGGAAAGATCCGT	CACTGATCACTCCCACACCC
Solyc01g110450.3.1	CACCCGAGATTGCCTAGCAT	TCCAGGCATGACACTGAACC
Solyc03g082560.3.1	ACGTCGGAAAACAGTACCACA	TGGAGAATCAAGAGCTGCGA
Solyc03g093270.3.1	CTTTGCTTGTGTCACTCCTGT	AACCACAGCAGTAGTTCCCC
Solyc03g093280.3.1	TGTGTCACTCCGGTTGTCAAT	TGGAGTTGCATTATGCTTTTGGG
Solyc03g093290.2.1	CATTGGCCAGTGGCTTTGAA	TGGATGCATTTCCACTCCGA
Solyc03g098100.4.1	GGAACTGCTGCGTATCCCAT	TGCCTCGGTCTATTGCTTCC
Solyc04g008440.1.1	TGGTGCACTTTTGCTCAACG	CCATACCTTCCCACACACCC
Solyc06g053600.4.1	GAGGCCAAGTGTGCCTTTTG	GCACCTAACCAGCAGACTGT
Solyc07g043570.3.1	GCCCAATGAGGGGTCAGAAA	AGCTTGTGTCACCCCAAGAC
Solyc09g011240.3.1	CAACAAGCCAAATTGCGGGA	TGAATGCCCCAACGAAGACA
Solyc09g015070.3.1	GGCAGAGTACGGAGTCCAAG	GAGACGGAAGGGCCAATGAA
Solyc09g074310.3.1	CACTGGCCTGATCGCTATGT	CCCGCTGTACTTTTGGCAAC
Solyc09g082720.3.1	AGTCTGAAGCGGCTTGATGT	TGAACAGCATGTGCCCTCTT
Solyc09g082730.5.1	ATCTGAAGCCAGCGTTGACA	TCTGTGATTGCCTTCCCACC
Solyc09g097950.3.1	CAGTTTACGGGCCACCCAA	TTTTTCCCTCTTTCGTCATCTTGC
Solyc09g097960.3.1	GTCCGATTTCTACGGTCCGC	TGCTAACTCGACTCGTTCCC
Solyc09g097970.3.1	TCCACCACGCCATTAACTCC	TCCGCGCACTTGCATTTTTC
Solyc09g097980.4.1	TCCACCACGCCATTAACTCC	GGCTGGATTTCCATGCACTT
Solyc09g097990.1.1	GCACCTACCAAGGTTCCAGG	ACAACACAGCTGCCATGGAT
Solyc09g098000.4.1	GCTGCTCGAGGATCTGTCAA	TAGTGGTACCTGGGATGGGG
Solyc09g098090.4.1	GTTGATTGAGAGCTTGGCCG	CTTGGTGGTACCGGGTATGG
Solyc11g067160.2.1	CGATTAAGTGTTGCTCGCCG	AAGCCCAGCGTCTGCATAAT
Solyc12g042470.2.1	TTGCATGGAGGCAGGAGAAG	CAACGCTCTTGCGTTTGTGA
Solyc12g098150.2.1	GGAGCATGGGTTACCTTTGGA	TTCACGAATCGCTTGACCCA

Table S6 Primers of silencing genes

Gene No.	Forward primer Sequence (5'-3')	Reverse primer Sequence (5'-3')
Solyc01g106450.3.1	ccggaattcCACTGGGCAACACTGGAC	cgcggatccTAACATATCAACATAATC
Solyc07g043570.3.1	ccggaattcGCATTTGAGCATTGGGTG	ccggaattcTCTTTCCTTGATGAATCT
Solyc09g011240.3.1	ccggaattcATGGCGGAGGCGACGGAG	ccggaattcCCGAGCCAGGTTTCATAC

Lowercase letters are sequences of restriction endonuclease sites, with foward :*Eco*RI and reverse:*Bam*HI

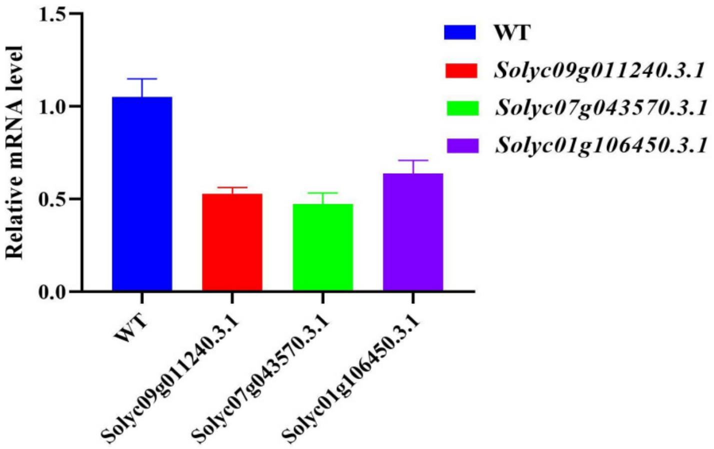

Figure S1 Silence efficiency of *Solyc09g011240*, *Solyc07g043570* and *Solyc01g106450* silenced plants.
